# The performance of Cobas HPV test for cervical cancer screening in Chinese female migrant workers

**DOI:** 10.1017/S0950268821001904

**Published:** 2021-08-09

**Authors:** Yaojuan He, Wenting Chen, Zhiliang Guo, Danni Yi, Chunfang Cai, Ying Shi, Yanfen Long, Kun Shi

**Affiliations:** Gynecologic Outpatient Department, Guangzhou Women and Children's Medical Center, Guangzhou 510621, P.R. China

**Keywords:** Cervical cancer, Cobas HPV test, human papillomavirus

## Abstract

This study aimed to evaluate the performance of Cobas human papillomavirus (HPV) test in cervical cancer screening. A total of 3442 women aged ⩾20 years used Cobas HPV and hybrid capture 2 (HC2) tests were included in this study. Women with any positive result were examined by liquid-based cytology (LBC) test. Then subjects with abnormal LBC or positive Cobas HPV16/18 were further checked by colposcopy to observe the visible lesions to perform the pathological examination. Of these 3442 women, 328 cases were Cobas HPV positive, and the positive rate was 9.53% (95% confidence interval (CI) 8.50–10.53). The positive rate of HPV16, HPV18, and other 12 types of high-risk HPV were 1.54% (95% CI 1.12–1.95), 0.55% (95% CI 0.30–0.80), and 7.44% (95% CI 6.56–8.32), respectively. The coincidence rate of Cobas HPV test and HC2 test was 90% (95% CI 89.00–91.00; Kappa = 0.526) in the primary screening. Age had a non-linear relationship with Cobas HPV positive rate (*χ*^2^ = 4.240, *P* = 0.040) and HPV16/18 typing positive rate (*χ*^2^ = 6.610, *P* = 0.010). Compared with the LBC test, the Cobas HPV test had higher sensitivity when detecting patients with high cervical intraepithelial neoplasia (CIN2+ and CIN3+).

## Introduction

Cervical cancer is the fourth most common cancer in women globally [1], with more than 500 000 new cases and 300 000 deaths worldwide every year [2]. The burden faced by developing countries is significantly greater than developed countries due to the lack of resources and infrastructure such as organised vaccination and screening programmes for cervical cancer [1]. There are about 130 000 new cases each year in China, accounting for 18.6% of the world's total new cases [3]. Persistent infection of high-risk human papillomavirus (HPV) is the main cause of cervical cancer [4]. HPV DNA can be detected in up to 99.7% of cervical cancer patients [5], making high-risk HPV detection an effective screening method for cervical cancer.

Based on the pathogenicity, the high-risk HPV genotypes were divided into 15 types, which were closely related to cervical cancer and cervical intraepithelial neoplasia (CIN). They were HPV16, 18, 31, 33, 35, 39, 45, 51, 52, 53, 56, 58, 59, 66 and 68, respectively [6]. Currently, it has been proven that HPV16 and 18 are the most virulent high-risk genotypes, accounting for about 70% of all invasive cervical cancer worldwide [7]. The main HPV screening technology used in China is hybrid capture 2 (HC2), which was the earliest HPV detection technology used in clinics and could detect 13 kinds of high-risk HPV (HPV16, 18, 31, 33, 35, 39, 45, 51, 52, 56, 58, 59 and 68). However, HPV16 and 18 could not be typed [8]. In clinical application, women with high-risk HPV16/18 positive and normal cytology could not get timely vaginoscopy referral [9]. The Cobas HPV test was a diagnostic technique *in vitro* for cervical cancer screening in recent years, which can detect 14 high-risk HPV subtypes, specifically report the results of high-risk HPV16 and 18 subtypes, and provide pooled results for the 12 other high-risk HPV subtypes (31, 33, 35, 39, 45, 51, 52, 56, 58, 59, 66 and 68) [10]. In this study, we aimed to compare the consistency between the Cobas HPV test and the HC2 test, in order to evaluate the performance of the Cobas HPV test technology in cervical cancer screening.

## Methods

### Study design and population

This is a cross-sectional analysis, and a total of 7004 women were recruited into the Chinese public welfare project for two types of cancer screening (cervical cancer and breast cancer, 2015) in Guangzhou Women and Children's Medical Center from December 2016 to June 2018. All of the participants were migrant workers, who lack regular cervical cancer screening. Before the HPV test, a questionnaire was conducted on the subjects’ education, marriage, contraceptive methods and cognition of cervical cancer. Participants were included if they met the following inclusion criteria: (1) subjects received both Cobas HPV test and HC2 test; (2) age ⩾20 years and (3) subjects with a history of sexual life, non-pregnancy, no history of cervical surgery. This study was approved by the Institutional Review Board (IRB) of the Guangzhou Women and Children's Medical Center, and written informed consent was obtained from all participants.

### Screening procedures

Established files and registers, and provided informed consent and epidemiological investigations for women who met the screening conditions and voluntarily accepted the screening, and carried out Cobas HPV test and HC2 test (the primary screening). The positive results were detected by the liquid-based cytology (LBC) test (the second screening), and those with cytological abnormities or positive Cobas HPV16/18 were referred to colposcopy for examination. If necessary, a cervical biopsy was performed under colposcopy. The pathological diagnosis was performed by qualified pathologists in Guangzhou Women and Children's Medical Center. The accuracy of the screening methods was evaluated by pathologically confirmed cases of CIN2 or above.

### HC2 test

HC2 was a nucleic acid hybridisation detection method using microplate chemiluminescence for signal amplification, which could detect 13 types of high-risk HPV DNA (HPV16, 18, 31, 33, 35, 39, 45, 51, 52, 56, 58, 59 and 68) at one time by 96-well plate method, and simultaneously detect the viral load of HPV DNA in the samples. The HC2 test was performed by using the HC2 sample conversion kit (Qiagen) for the PreservCyt medium. If the relative light unit/cutoff ratio of the sample was ⩾1.0, the sample was recorded as positive.

### Cobas HPV test

Cobas HPV test used the same specimens as the LBC test. Cobas HPV test is an *in-vitro* quantitative detection technique for high-risk HPV DNA by polymerase chain reaction amplification, and can detect HPV16, 18 and other 12 types of high-risk HPV types (31, 33, 35, 39, 45, 51, 52, 56, 58, 59, 66 and 68), respectively. The Cobas HPV test was carried out according to the manufacturer's protocol [11]. Interpretation of the amplification and detection stage was carried out using software supplied with the Cobas 4800 platform.

### LBC test

The diagnostic criteria of the Bethesda System (TBS, 2001) [12], LBC and sedimentation thin-layer methods were used for the LBC test. The reports were interpreted for negative for intraepithelial lesion or malignancy (NILM), atypical squamous cells of undetermined significance (ASCUS), atypical squamous cells cannot exclude HSIL (ASC-H), low-grade squamous intraepithelial lesion (LSIL) and high-grade squamous intraepithelial lesion (HSIL). The diagnosis result of NILM is considered normal, and the positive result of any one of ASCUS, ASC-H, LSIL and HSIL is considered abnormal.

### Colposcopy test and cervical biopsy

Five percent acetic acid solution was applied on the surface of the cervix. After 1 min, the changes in cervix were observed under a colposcope. Preliminary diagnosis was made according to the thickness, extent, surface morphology and turbidity of the acetowhite epithelium. Healthy cervix had no acetowhite epithelium. LSIL included HPV infection and CIN1, which has a light acetowhite epithelium and could be at or outside the junction of squamous column. HSIL included CIN2, CIN3 and cervical carcinoma *in situ*. HSIL was characterised by thick acetowhite epithelium with clear boundary and was located near the junction of squamous column. Invasive carcinoma of cervix was featured with irregular, thick and brittle masses on the surface of the acetowhite epithelium. Cervical biopsy was performed under a colposcope. If lesions were visible under the colposcope, biopsy was taken directly at the lesion site, and if there were no visible lesions, biopsy was not performed.

### Statistical analysis

SAS 9.4 software (SAS Institute, Inc., Cary, North Carolina) was used for statistical analysis. Quantitative variables were tested by the *t*-test and expressed as mean ± s.d. Categorical variables were analysed by the chi-squared test (*χ*^2^ test) or the Fisher's exact test and displayed as number (*n*) and percentage (%). The Cohen's kappa was calculated to compare the HC2 test and Cobas HPV test results.

When evaluating the screening efficacy of the Cobas HPV test and LBC test in detecting HSIL, pathological diagnosis was used as the gold standard and CIN2+ and CIN3+ were the disease endpoints respectively. MedCalc 14.8.1 software (Medcalc Software bvba, Ostend, Belgium) was used to calculate the sensitivity, specificity, positive-predictive value, and negative-predictive value (NPV) of Cobas HPV test and LBC test. All statistical analyses were the two-sided test. The 95% confidence intervals (95% CIs) were calculated, and *P* < 0.05 was considered statistically significant.

## Results

### Baseline characteristics

**Fig. 1.**
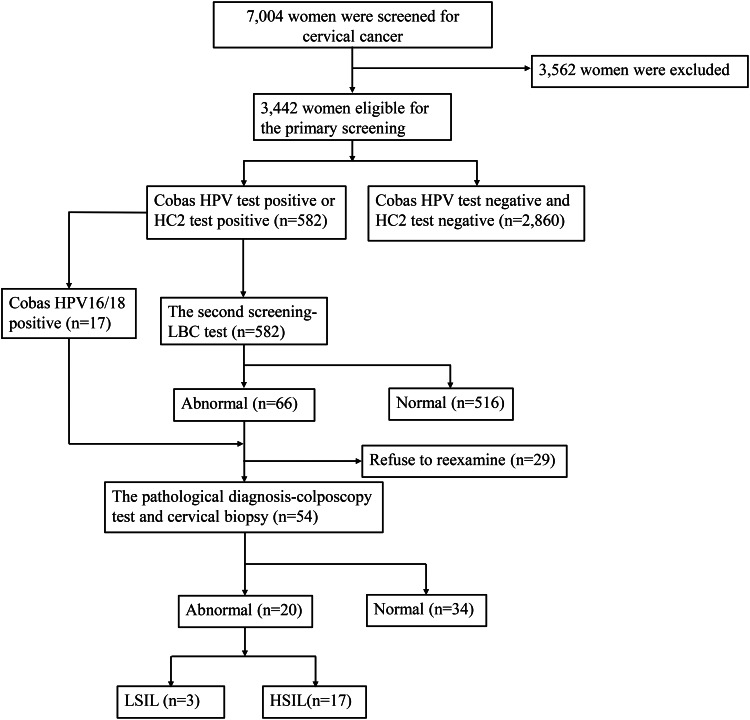
Flowchart of the screening profile.

A total of 3442 women met the inclusion criteria (Fig. 1). The mean age of subjects was 42.23 ± 8.16 years. Of these women, 3270 were the Han nationality (95.00%), 723 were primary school education level or illiterate (21.00%), 2255 graduated from junior middle schools or senior high schools (65.50%) and 464 from junior colleges or above (13.50). Their educational levels were concentrated in the lower educational levels. Among them, 2882 were married (82.00%), 327 were unmarried and had sex (9.5%) and 293 were divorced, separated or widowed (8.5%). In total, 2168 participants had never received a formal gynaecological examination (62.99%), and 585 had received cervical HPV or LBC tests in the past 3 years (17.00%) (Table 1).

**Table 1.** Baseline characteristics of the subjects

Variables	Data (*n* = 3442)
Age (years), mean ± s.d.	42.23 ± 8.16
Nationality, *n* (%)	
Han	3270 (95.00)
Others	175 (5.00)
Education level, *n* (%)	
Primary or illiterate	723 (21.00)
Junior high or high school	2255 (65.50)
Junior colleges or above	464 (13.50)
Marital status, *n* (%)	
Married	2882 (82.00)
Unmarried (had sex)	327 (9.50)
Divorced, separated or widowed	293 (8.50)
Formal gynaecological examination, *n* (%)	
No	2168 (62.99)
Yes	1274 (37.01)
Cervical HPV or LBC tests (past 3 years), *n* (%)	
No	2857 (83.00)
Yes	585 (17.00)

### Comparison of Cobas HPV test and HC2 test (the primary screening)

Totally, 328 cases were Cobas HPV positive, and the positive rate was 9.53% (95% CI 8.50–10.53). The positive rate of HPV16, HPV18 and other 12 types of high-risk HPV were 1.54% (95% CI 1.12–1.95), 0.55% (95% CI 0.30–0.80) and 7.44% (95% CI 6.56–8.32), respectively. A total of 492 cases were found to be HC2 positive, and the positive rate was 14.29% (95% CI 13.09–15.51). The coincidence rate of Cobas HPV test and HC2 test was 90% (95% CI 89.00–91.00), which is medium-high consistency and statistically significant (Kappa = 0.526, *P* < 0.001) (Tables 2 and 3).

**Table 2.** Screening results of the Cobas HPV test and HC2 test in different age groups

Methods	Age groups (years)					Total (*n* = 3442)
	<30 (*n* = 233)	30–39 (*n* = 990)	40–49 (*n* = 1670)	50–59 (*n* = 466)	⩾60 (*n* = 83)	
Cobas HPV, *n* (%)						
12hrHPV	25 (10.73)	74 (7.47)	117 (7.01)	34 (7.30)	6 (7.23)	256 (7.44)
HPV16	5 (2.15)	15 (1.52)	23 (1.38)	9 (1.93)	1 (1.20)	53 (1.54)
HPV18	4 (1.72)	5 (0.51)	6 (0.36)	2 (0.43)	2 (2.41)	19 (0.55)
Negative	199 (85.41)	896 (90.51)	1524 (91.26)	421 (90.34)	74 (89.16)	3114 (90.47)
HC2, *n* (%)						
Positive	44 (18.88)	146 (14.75)	225 (13.47)	63 (13.52)	14 (16.87)	492 (14.29)
Negative	189 (81.12)	844 (85.25)	1445 (86.53)	403 (86.48)	69 (83.13)	2950 (85.71)

**Table 3.** Comparison between Cobas HPV test and HC2 test

Cobas HPV	HC2 test		Total
	Positive	Negative	
Positive	238	90	328
Negative	254	2860	3114
Total	492	2950	3442
Kappa values	0.526		
*Z*	31.697		
*P*	<0.001		

**Fig. 2.**
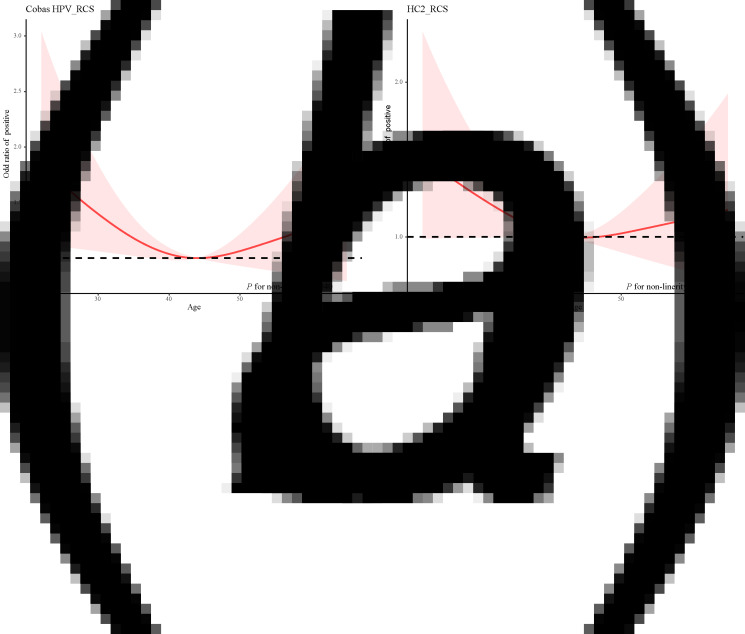
Relationship between age and Cobas HPV positive rate and HC2 positive rate. (a) The Cobas HPV positive rate and age and (b) The HC2 positive rate and age.

The restricted cubic spline was used to develop a model and visualise the relationship between age and Cobas HPV positive rate and HC2 positive rate. The results showed that there was a non-linear relationship between age and Cobas HPV positive rate (*χ*^2^ = 4.240, *P* = 0.040). A ‘U-shaped’ distribution was shown in the relationship between age and Cobas HPV positive rate, and there were two peak age groups, 20–29 years old and 60+ years old respectively. There may be a non-linear relationship between age and HC2 positive rate, but the non-linear test was not statistically significant (*P* = 0.143) (Fig. 2).

**Fig. 3.**
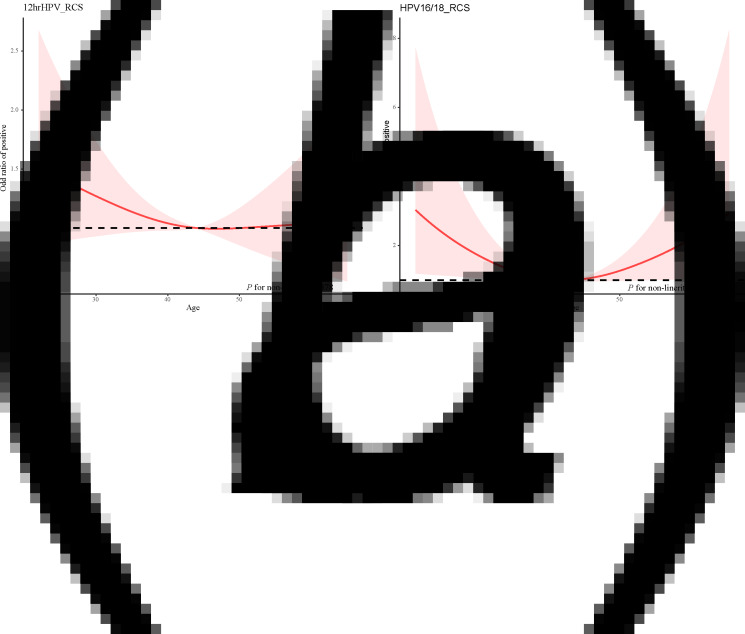
Relationship between age and Cobas HPV typing positive rate. (a) The 12hrHPV positive rate and age and (b) The HPV16/18 positive rate and age.

Similarly, the restricted cubic spline was used to develop a model and visualise the relationship between age and Cobas HPV typing positive rate. There was a non-linear relationship between age and HPV16/18 typing positive rate (*χ*^2^ = 6.610, *P* = 0.010). The age and HPV16/18 typing positive rate was a ‘U-shaped’ distribution, and there were two peak age groups, 20–29 years old and 60+ years old respectively. In addition, there may be a non-linear relationship between age and other 12 types of high-risk HPV typing positive rate, but the non-linear test was not statistically significant (*P* = 0.378) (Fig. 3).

### The results of LBC test (the second screening)

Of these 582 positive cases in the first screening, 66 cases were also positive in the LBC test (11.34%; 95% CI 8.76–13.92). The positive rate of ASC-US, LSIL, AGC, ASC-H and HSIL in the LBS test were 4.46% (95% CI 2.78–6.14), 1.89% (95% CI 0.78–3.00), 1.03% (95% CI 0.21–1.85), 2.03% (95% CI 0.88–3.18) and 1.72% (95% CI 0.66–2.78), respectively. The distribution of Cobas HPV positive rate in NILM, ASC-US, LSIL, AGC, ASC-H and HSIL were 46.38% (95% CI 42.33–50.43), 3.96% (95% CI 2.38–5.54), 1.89% (95% CI 0.78–3.00), 0.51% (95% CI 0.0–1.09), 2.06% (95% CI 0.91–3.21) and 1.52% (95% CI 0.53–2.51), respectively. The detailed results were shown in Tables 4 and 5.

**Table 4.** Screening results of the LBC test in different age groups

LBC, *n* (%)	Age groups (years)					Total (*n* = 582)
	<30 (*n* = 52)	30–39 (*n* = 172)	40–49 (*n* = 265)	50–59 (*n* = 78)	⩾60 (*n* = 15)	
NILM	46 (88.46)	155 (90.12)	236 (89.06)	66 (84.62)	13 (86.67)	516 (88.66)
ASC-US	4 (7.69)	6 (3.49)	11 (4.15)	5 (6.41)	1 (6.67)	27 (4.46)
LSIL	1 (1.92)	3 (1.74)	5 (1.89)	1 (1.28)	1 (6.67)	11 (1.89)
AGC	0 (0.00)	1 (0.58)	3 (1.13)	2 (2.56)	0 (0.00)	6 (1.03)
ASC-H	1 (1.92)	3 (1.74)	4 (1.51)	4 (5.13)	0 (0.00)	12 (2.03)
HSIL	0 (0.00)	4 (2.33)	6 (2.26)	0 (0.00)	0 (0.00)	10 (1.72)

**Table 5.** Positive rate of Cobas HPV and Cobas HPV typing in different LBC test indicators

Cobas HPV typing, *n* (%)	LBC test						Total (*n* = 582)
	NILM	ASC-US	LSIL	AGC	ASC-H	HSIL	
14hrHPV	270 (46.38)	23 (3.96)	11 (1.89)	3 (0.51)	12 (2.06)	9 (1.52)	328
12hrHPV+	222 (38.14)	14 (2.41)	11 (1.89)	1 (0.17)	4 (0.69)	4 (0.69)	256
HPV16	35 (6.01)	5 (0.86)	0 (0.00)	1 (0.17)	8 (1.37)	4 (0.69)	53
HPV18	13 (2.23)	4 (0.69)	0 (0.00)	1 (0.17)	0 (0.00)	1 (0.14)	19
HPV16/18	48 (8.24)	9 (1.55)	0 (0.00)	2 (0.34)	8 (1.37)	5 (0.83)	72

### The results of colposcopy test and cervical biopsy (the pathological diagnosis)

Among 54 positive cases after two screenings, 20 cases were positive (37.04%; 95% CI, 24.16–49.92) according to the pathological diagnosis. The incidence rates of CIN1, CIN2, CIN3 and squamous/adenocarcinoma were 5.56% (95% CI 0.0–11.73), 12.96% (95% CI 4.00–21.92), 11.12% (95% CI 2.73–19.51) and 7.40% (95% CI 0.42–14.38), respectively (Table 6).

**Table 6.** Results of the pathological diagnosis in different age groups

The pathological diagnosis, *n* (%)	Age group (years)					Total (*n* = 54)
	<30 (*n* = 7)	30–39 (*n* = 13)	40–49 (*n* = 23)	50–59 (*n* = 11)	⩾60 (*n* = 0)	
HPV infection	7 (100.00)	8 (61.54)	13 (56.52)	6 (54.55)	0 (0.00)	34 (62.96)
CIN1	0 (0.00)	1 (7.69)	1 (4.35)	1 (9.09)	0 (0.00)	3 (5.56)
CIN2	0 (0.00)	2 (15.38)	5 (21.74)	0 (0.00)	0 (0.00)	7 (12.96)
CIN3	0 (0.00)	1 (7.69)	3 (13.04)	2 (18.18)	0 (0.00)	6 (11.12)
Squamous or adenocarcinoma	0 (0.00)	1 (7.69)	1 (4.35)	2 (18.18)	0 (0.00)	4 (7.40)

### Comparison of the efficacy of Cobas HPV test and LBC test in detecting HSIL

When CIN2+ is used as the disease endpoint to compare the efficacy of the two tests, the sensitivity ((76.5%; 95% CI 50.1–93.2) *vs.* (29.4%; 95% CI 10.3–56.0), *P* = 0.002) and NPV ((85.2%; 95% CI 70.2–93.4) *vs.* (75.0%; 95% CI 68.7–80.4), *P* = 0.042) were higher in the Cobas HPV test than those in the LBC test. Similarly, when CIN3+ is used as the disease endpoint, compared with the LBC test, the sensitivity ((80.0%; 95% CI 44.4–97.5) *vs.* (30.0%; 95% CI 6.7–65.2), *P* = 0.009) and NPV ((92.6%; 95% CI 77.9–97.8) *vs.* (85.4%; 95% CI 79.5–89.9), *P* = 0.315) were also higher in the Cobas HPV test. The Cobas HPV test is more effective at detecting CIN2+ and above than the LBC test (Tables 7 and 8).

**Table 7.** Distribution of CIN2+ and CIN3+ detected by the Cobas HPV test and the LBC test

Screening	The pathological diagnosis			
	CIN2+		CIN3+	
Cobas HPV	+	−	+	−
+	13	14	8	19
−	4	23	2	25
LBC				
+	5	1	3	3
−	14	36	7	41

**Table 8.** Comparison of the efficacy of Cobas HPV test and LBC test in detecting CIN2+ and CIN3+

Variables	CIN2+		*Z*	*P*	CIN3+		*Z*	*P*
	Cobas HPV	LBC			Cobas HPV	LBC		
Sensitivity	76.5% (50.1–93.2)	29.4% (10.3–56.0)	3.118	0.002	80.0% (44.4–97.5)	30.0% (6.7–65.2)	2.599	0.009
Specificity	62.2% (44.8–77.5)	97.3% (85.8–99.9)	4.176	<0.001	56.8% (41.0–71.7)	93.2% (81.3–98.6)	4.345	<0.001
PPV	48.2% (36.3–60.3)	83.3% (38.7–97.5)	1.949	0.051	29.6% (21.0–40.0)	50.0% (19.1–80.9)	0.918	0.359
NPV	85.2% (70.2–93.4)	75.0% (68.7–80.4)	2.034	0.042	92.6% (77.9–97.8)	85.4% (79.5–89.9)	1.005	0.315

PPV, positive predictive value; NPV, negative predictive value.

## Discussion

In this study, we evaluated the performance of Cobas HPV test for cervical cancer screening in female migrant workers. The positive rate detected by the Cobas HPV test was 9.53%, and the coincidence rate of the Cobas HPV test and HC2 test was 90% in the primary screening. The Cobas HPV positive rate and HPV16/18 typing positive rate had a non-linear relationship with age. A ‘U-shaped’ distribution was shown in the relationship between age and Cobas HPV positive rate and HPV16/18 typing positive rate, and there were two peak age groups, 20–29 years old and 60+ years old respectively. Compared with the LBC test, the Cobas HPV test had higher sensitivity and NPV when detecting patients with CIN2+ and CIN3+.

Persistent high-risk HPV infection is one of the leading causes of cervical carcinoma and related deaths in women worldwide. It was reported that HPV16 and HPV18 in high-risk HPV types could cause about 70% of cervical squamous cell carcinomas and about 80% of cervical adenocarcinomas [13]. In China, HPV16 and HPV18 accounted for 76.7% and 7.8%, respectively [14]. In April 2014, FDA approved Roche's Cobas HPV test for first-line primary screening of cervical carcinoma [10]. However, the Cobas HPV test is not included in the routine first-line screening programme for cervical cancer in China. Our study compared the performance of Cobas HPV test and HC2 test in primary screening of cervical cancer. The results showed that the coincidence rate of the Cobas HPV test and HC2 test was 90% in the primary screening for cervical cancer. Similar results were performed in the researches of other scholars using Cobas HPV and HC2 test [15, 16]. In addition, the Cobas HPV positive rate and HPV16/18 typing positive rate had a U-shaped distribution with age were presented in our study, and there were two peak age groups, 20–29 years old and 60+ years old. Several studies have analysed the U-shaped functions in biological characteristics [17–19]. In HPV infection, some studies have found that there was a U-shaped distribution between HPV infection risk and age [20–22]. The results of these studies were consistent with ours. A more detailed study displayed that those women at the age of 20–29 years old had the highest prevalence of HPV infection and a second peak was observed at the age of ⩾60 years old [23]. The highest incidence and prevalence of infection with high-risk HPV types usually is observed in women aged <25 years and decreases with age [24, 25]. In addition, approximately one in five new cases of cervical cancer are diagnosed in women ⩾65 years, this phenomenon is largely attributable to a lack of screening [26, 27]. The U-shaped distribution between HPV infection risk and age suggested that cervical cancer screening may focus on people aged 20–29 and over 60.

Our study also compared the performance of Cobas HPV test and LBC test in detecting HSIL. The Cobas HPV test had higher sensitivity and NPV than the LBC test when detecting CIN2+ and CIN3+ patients. A prospective study performed that the Cobas HPV test had higher sensitivity than the LBC test in detecting CIN2+ and CIN3+ lesions, and the combination use of Cobas HPV test and LBC test can improve specificity [28]. Dreyer *et al*. showed that the best outcomes for detection of disease were seen using the Cobas HPV test compared with the LBC test [29].

In this study, most women who participated in cervical cancer screening were married women who made a living by labour work in the city. Most of them were in the service industry and lacked the awareness of active physical examinations [30]. In addition, their compliance with medical advice was low, and the lost to follow-up rate was very high. Therefore, to obtain the most information from one tissue sample was a better screening plan. Cobas HPV screening using LBC specimens could achieve simultaneous detection of virus and cells in one sample, reducing sampling frequencies and the difficulty of follow-up. Besides, the samples of Cobas HPV test can last for 1 month without losing test performance compared to the LBC sample [31]. Furthermore, Cobas HPV16/18 positive patients can be referred to colposcopy as soon as possible [32], which can reduce the number of return visits and avoid the possibility of missed diagnosis caused by negative results of LBC.

## Conclusion

The Cobas HPV test was more sensitive than LBC in the application of cervical cancer screening, especially in a large-scale population with a high potential infection rate. Compared with the HC2 test, Cobas HPV reported the results of HPV16 and 18 while detecting the high-risk HPV, which is helpful for doctors to refer high-risk groups to colposcopy in time, and to detect and treat patients with high risks of pathological changes earlier.

## Data Availability

The datasets used and/or analysed during the current study are available from the corresponding author on reasonable request.
